# Prognostic significance of microRNA-101 in solid tumor: A meta-analysis

**DOI:** 10.1371/journal.pone.0180173

**Published:** 2017-07-25

**Authors:** Xianxiong Ma, Jie Bai, Gengchen Xie, Yulin Liu, Xiaoming Shuai, Kaixiong Tao

**Affiliations:** Department of Gastrointestinal Surgery, Union Hospital, Tongji Medical College, Huazhong University of Science and Technology, Wuhan, Hubei Province, People’s Republic of China; University of Toronto, CANADA

## Abstract

MicroRNA-101 has been reported as an important factor in carcinogenesis of several malignant tumors. However, its actual role in prognosis among solid malignancies remains unclear. Accordingly, we performed this meta-analysis aiming to identify prognostic significance of miR-101 in solid tumor. Pooled hazard ratios (HRs) with 95% confidence intervals (CIs) for overall survival (OS) or disease-free survival (DFS)/metastasis-free survival (MFS)/progression-free survival (PFS)/relapse-free survival (RFS)/time-to progression (TTP) were estimated with random effects or fixed effects models on the basis of heterogeneity. Subgroup analysis, sensitive analysis and meta-regression analysis were also conducted to clarify the possible confounding factors and investigate the source of heterogeneity. Publication bias was evaluated by using Begg’s and Egger’s tests. A total of 21 studies containing 3753 cases were selected into our quantitative analysis via electronic database search. A lower expression of miR-101 was significantly associated with worse OS (HR = 0.66, 95%CI [0.52–0.85], P = 0.001) and PFS (HR = 0.70, 95%CI [0.51–0.95], P = 0.023) in patients with solid tumor. The under-expression of miRNA-101 is a credible indicator of poorer prognosis in several of solid malignancies.

## Introduction

MicroRNAs (miRNA, miRs) are a subset of small non-coding RNA molecules that are approximately 18–22 nucleotides in length. MiRNAs play crucial regulatory roles in gene expression at the post-transcriptional level [1, 2]. The major mechanism of miRNA action is the interaction with the 3’-UTR of the targeted gene mRNA, followed by degradation of the mRNA or inhibition of mRNA protein translation. In human cancers, numerous studies have shown that the expression of miRNAs is deregulated and these miRNAs act as regulatory molecules in many biological processes, including differentiation, proliferation, and apoptosis of tumor cells [3–5]. Several miRNAs are downregulated in many tumors and appear to function as tumor suppressor genes [6]. Among these downregulated miRNAs, miR-101 is one of the most downregulated miRNAs in human cancers, multiple research studies have been exploring the prognostic function of miR-101 in cancer patients in order to find a reliable biomarker to guide for cancer treatment[7, 8].

MiRNA-101, located on chromosome 1[65058434–65058508], is widely known as a tumor suppressive miRNA that is strongly downregulated in several cancers including neuroblastoma, gastric cancer, prostate cancer, nasopharyngeal carcinoma and hepatocellular carcinoma [9–11]. The aberrant expression of miR-101 not only has diagnostic implications but also can predict cancer patient survival [12]. Although an overwhelming majority of evidence has explored a negative prognostic value of miR-101 under-expression across miscellaneous neoplasms, the prognostic impact of miR-101 in malignancies remains controversial. Failed to draw a similar conclusion, Slattery et al[13] and Lv et al [14] presented a worse survival status of patients under stronger miR-101 expression, suggesting an astonishing positive prognostic significance in circumstance of miR-101 under-expression. Therefore, in the present study through gathering available evidence, we carried out an integrated meta-analysis as well as subgroups analysis to identify the relationship between miR-101 expression level and survival of cancer patients by pooling the hazard ratio (HR) from studies addressing the correlation between miR-101 and OS/PFS of patients with malignancies, aiming to provide more theoretical supports for targeted treatment.

## Materials and methods

### Search strategy

We performed a thorough search for available literatures in electronic databases of Pubmed, Embase and Web of science until April 2017, using the following words “(microRNA-101 OR miR-101 OR miR101 OR miRNA-101) AND (tumor OR neoplasm OR cancer OR carcinoma OR malignancy)”. In order to avoid missing the potentially related articles, reference lists were also screened. Two authors independently carried out this procedure and any discrepancy was resolved by mutual discussion.

### Selection criteria

Inclusion criteria were as follows:1) studies exploring any of the solid tumor; 2) studies dealing with miR-101 expression and OS/DFS/PFS/RFS/MFS/TTP; 3) studies that categorized patients into low- and high-expression groups based on the miR-101 expression; 4) studies providing HR directly or key information to calculate HR indirectly, such as Kaplan-Meier curves and original survival data; 5) studies assessing miR-101 expression in tissue or blood.

The following were the exclusion criteria: 1) studies on myelomas, lymphomas, or leukemia; 2) duplicated or overlapped studies; non-original articles, such as reviews, articles or letters; 3) laboratory studies on cell lines or animals level; 4) studies on a set of microRNAs but not miR-101 alone; 5) studies with a sample-size less than 20 participants.

### Qualitative assessment

Newcastle-Ottawa Scale(NOS)[15] was adopted to evaluate the quality of each eligible article. The scale was revised with certain adaptive modifications to match the practical needs of the pooled analysis. There are three aspects contained in the scale: selection, comparability, and outcome. Stars awarded for each quality item serve as a quick visual assessment. Stars are awarded such that the highest quality studies are awarded up to nine stars. Studies with more than 6 stars were considered as of high quality. Otherwise, studies were excluded from the final meta-analysis.

### Data extraction

All eligible publications were reviewed by Shuai and Bai, The following details of each article were recorded: first author’s name, publication year, cancer type, treatment, sample size, stage of disease, miR-101 test method, the cutoff value to discriminate high or low expression of miR-101, sample sources, follow-up time, extracting method of HR, outcome, NOS and et al. The HR value was extracted directly if it was calculated by a multivariate analysis. Otherwise, the results from univariate analysis were also allowed in the meta-analysis. If both multivariate analysis and univariate analysis were not available, Kaplan-Meier curves were used to extract HR value by using the described method[16].

### Statistical analysis

The heterogeneity of the studies included in this meta-analysis was assessed by the Q statistic test and the I^2^ statistic test, where I^2^ more than 50% indicated evidence of heterogeneity[17]. The random-effects model was selected when I^2^ was significant (>50%); otherwise, the fixed-effects model was selected. Publication bias was examined using Begg’s funnel plot and Egger’s linear regression test. P<0.05 was considered significant[18]. All analyses were performed with STATA version 12.0 software (Stata Corporation, College Station, TX).

## Results

### Study selection

**Fig 1:**
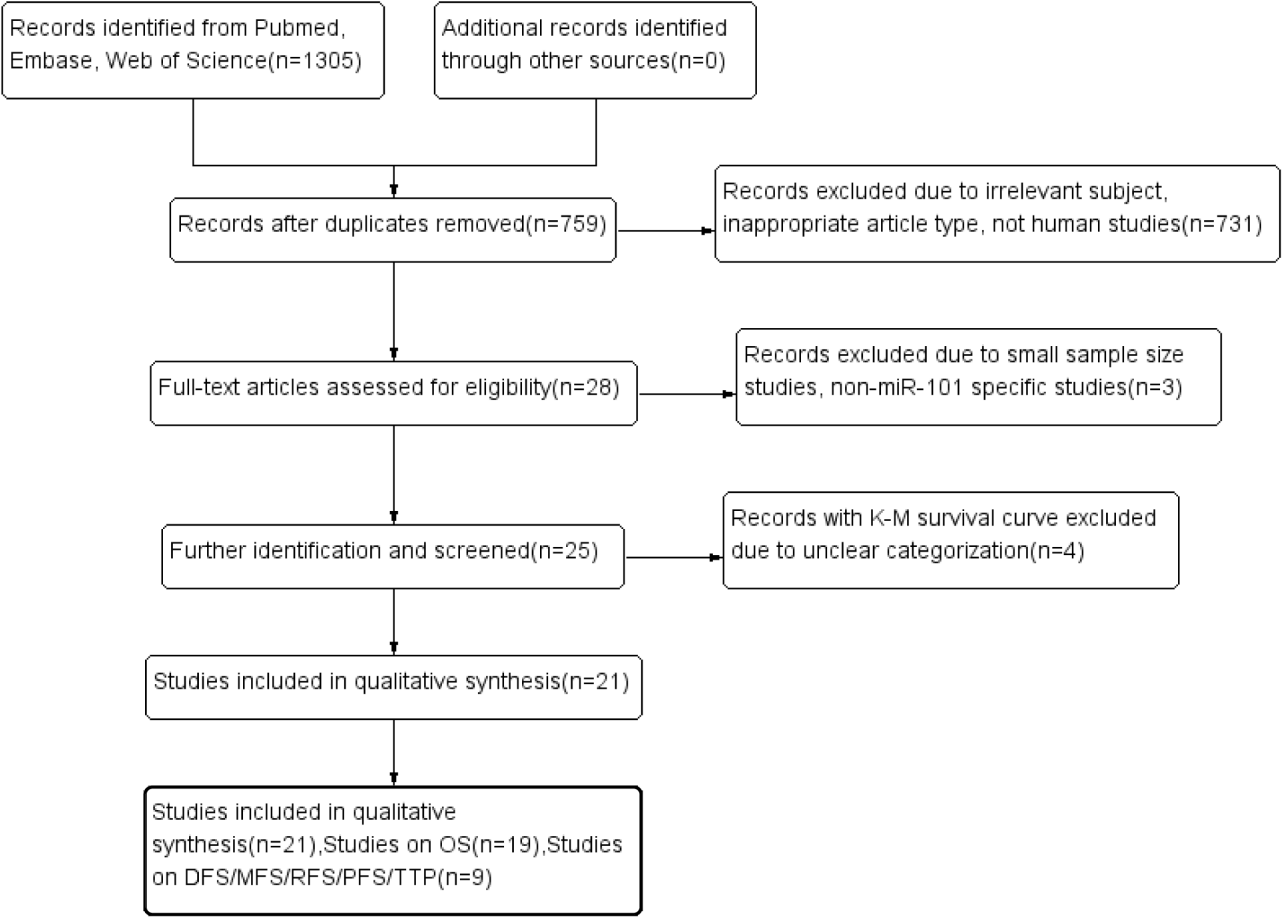
Flow diagram shows search strategy.

In total, 291, 331, 683 records were identified from Pubmed, Web of Science and Embase. According to the selection criteria, most of the preliminarily included entries were eliminated on account of duplicated data, inappropriate article type or inadequate original information. Finally, a total of 21[13, 14, 19–37] observational studies consisting of 3753 cases were retained for subsequent pooling calculation. None of the eligible entries scored less than six by NOS. **Fig 1**displayed the selection workflow of all eligible studies in our meta-analysis.

### Characteristics of included studies

The majority of included studies were carried out in China (n = 15), the other six studies were conducted in Netherlands (n = 2), USA, Japan, India, and Norway. None of the eligible entries scored less than six by NOS, indicating a high methodological quality across all studies. The cancer types included HCC, BTCC, NSCLC, GBC, CRC, LSCC, GBM, ESAC, PDAC, astrocytoma and glioma. Four study clearly stated the research-related treatment, as shown in **Table 1**, three studies did not clarify whether patient received adjuvant therapy after surgery, most of studies (n = 14) did not receive any adjuvant treatment after surgery. Study sample sizes ranged from 21 to 1134, qRT-PCR (n = 19) and microarray (n = 2) were used to assess miR-101 expression, and cutoff value varied among studies with median expression of miR-101 the most widely used. 15 studies enrolled patients with stages Ⅰ-Ⅳ and five studies explored with stages Ⅰ-Ⅲ (n = 4) or stages Ⅲ-Ⅳ (n = 1), only one study did not specify the stage of disease in the study population. The source of miR-101 came from tissue (n = 20) and blood (n = 1). 13 HRs were reported in the present analysis. The other 8 HRs were estimated by analyzing K-M curves. About a half of HRs (n = 11) were calculated by using a multivariate analysis and the remaining 10 records either did not clarify the calculating methods or were computed by using univariate analysis. 19 studies provided data on OS and 9 studies provided on DFS/PFS/RFS/MFS/TTP with respect to outcome.

**Table 1:** Characteristics of the included articles.

Study	Country	Cancer type	Treatment	NO. of patients	Stage	Test method	Cutoff	Sample source	Follow-up (months)	Extracting method	Multiple analysis	Male/female	Mean age	Outcome	NOS
Zheng 2015^17^	China	HCC	Surgery	163	Ⅰ-Ⅳ	qRT-PCR	ROC^B^	Blood	70	Report	Yes	136/27	NA	OS	8
Zhang 2012^18^	China	HCC	Surgery	130	Ⅰ-Ⅳ	qRT-PCR	Median	Tissue	8.6y^E^	Report	Yes	96/34	NA	OS/DFS	9
Zhang 2014^19^	China	BTCC	Surgery	72	Ⅰ-Ⅳ	qRT-PCR	1.45N^C^	Tissue	every 3m	Report	Yes	42/30	57	OS	8
Ye 2016^20^	China	NSCLC	NA	105	Ⅰ-Ⅳ	qRT-PCR	Median	Tissue	NA	Report	Yes	67/38	NA	OS	8
Lv 2016^12^	China	HCC	Surgery	78	Ⅰ-Ⅲ	qRT-PCR	Average	Tissue	60	Report	Yes	63/15	53	OS/RFS	9
Chandra 2017^21^	India	BC	NA	37	Ⅰ-Ⅳ	qRT-PCR	Median	Tissue	NA	Report	No	NA	NA	OS/MFS	8
Bao 2016^22^	China	GBC	Surgery	53	Ⅰ-Ⅳ	qRT-PCR	NA	Tissue	40	Report	Yes	18/35	NA	OS	8
Shen 2013^23^	China	HCC	NA	154	NA	Microarray	NA	Tissue	NA	K-M	No	NA	NA	OS	7
Slattery 2016^11^	USA	CRC	Surgery	1134	Ⅰ-Ⅳ	Microarray	NA	Tissue	60.4	Report	No	597/537	65.4	OS	8
Li J 2015^24^	China	BC	Surgery	111	Ⅰ-Ⅲ	qRT-PCR	NA	Tissue	NA	K-M	No	NA	NA	OS/DFS	7
Li M 2015^25^	China	LSCC	Surgery	80	Ⅰ-Ⅳ	qRT-PCR	Median	Tissue	60	K-M	No	56/24	NA	OS	7
Li X 2013^26^	China	Gliomas	Surgery	50	Ⅰ-Ⅳ	qRT-PCR	8^D^	Tissue	30	K-M	No	34/16	41	OS	7
Tian 2016^27^	China	GBM	S+R+C^A^	70	Ⅰ-Ⅳ	qRT-PCR	Average	Tissue	NA	K-M	No	33/37	NA	OS/PFS	7
Gao 2015^28^	China	CRC	Surgery	735	Ⅰ-Ⅳ	qRT-PCR	NA	Tissue	56(m)^F^	Report	Yes	NA	NA	OS/DFS	8
Hiroki 2009^29^	Japan	ESAC	Surgery	21	Ⅰ-Ⅳ	qRT-PCR	NA	Tissue	23(m)	Report	Yes	NA	64.9(m)	OS/DFS	8
Schee 2012^32^	Norway	CRC	Surgery	193	Ⅰ-Ⅲ	qRT-PCR	Median	Tissue	NA	K-M	No	112/81	NA	MFS	7
Maftouh M 2014^33^	Netherlands	PDAC	S+C	25	Ⅲ-Ⅳ	qRT-PCR	Median	Tissue	NA	K-M	No	NA	NA	OS	6
Liu C 2017^34^	China	Astrocytoma	S+R+C	80	Ⅰ-Ⅳ	qRT-PCR	NA	Tissue	NA	Report	Yes	36/54	NA	OS	7
Luo L 2011^35^	China	NSCLC	Surgery	45	Ⅰ-Ⅲ	qRT-PCR	0.54	Tissue	NA	K-M	No	29/16	NA	OS	7
Zhang S 2015^36^	China	CRC	Surgery	172	Ⅰ-Ⅳ	qRT-PCR	Median	Tissue	NA	Report	Yes	99/73	NA	OS	8
Jansen 2012^37^	Netherlands	BC	S+C	245	Ⅰ-Ⅳ	qRT-PCR	Median	Tissue	89(10–165)	Report	Yes	0/235	NA	TTP	8

Note

**A:** surgery + radiotherapy + chemotherapy

**B:** Categorized based on receiver operating curve

**C:** based on 1.45 fold of normal expression

**D:** did not state the definition of cutoff

**E:** 8.6Years

**F:** median follow-up.

**Abbreviations:** HCC hepatocellular carcinoma; BTCC bladder transitional cell carcinoma; NSCLC non-small-cell lung cancer; BC breast cancer; CRC colorectal cancer; GBC gallbladder carcinoma; LSCC laryngeal squamous cell carcinoma; GBM glioblastoma multiforme; ESAC endometrial serous adenocarcinoma; PDAC pancreatic ductal adenocarcinoma.

### Correlation of miR-101 expression with OS and subgroup analysis

**Fig 2:**
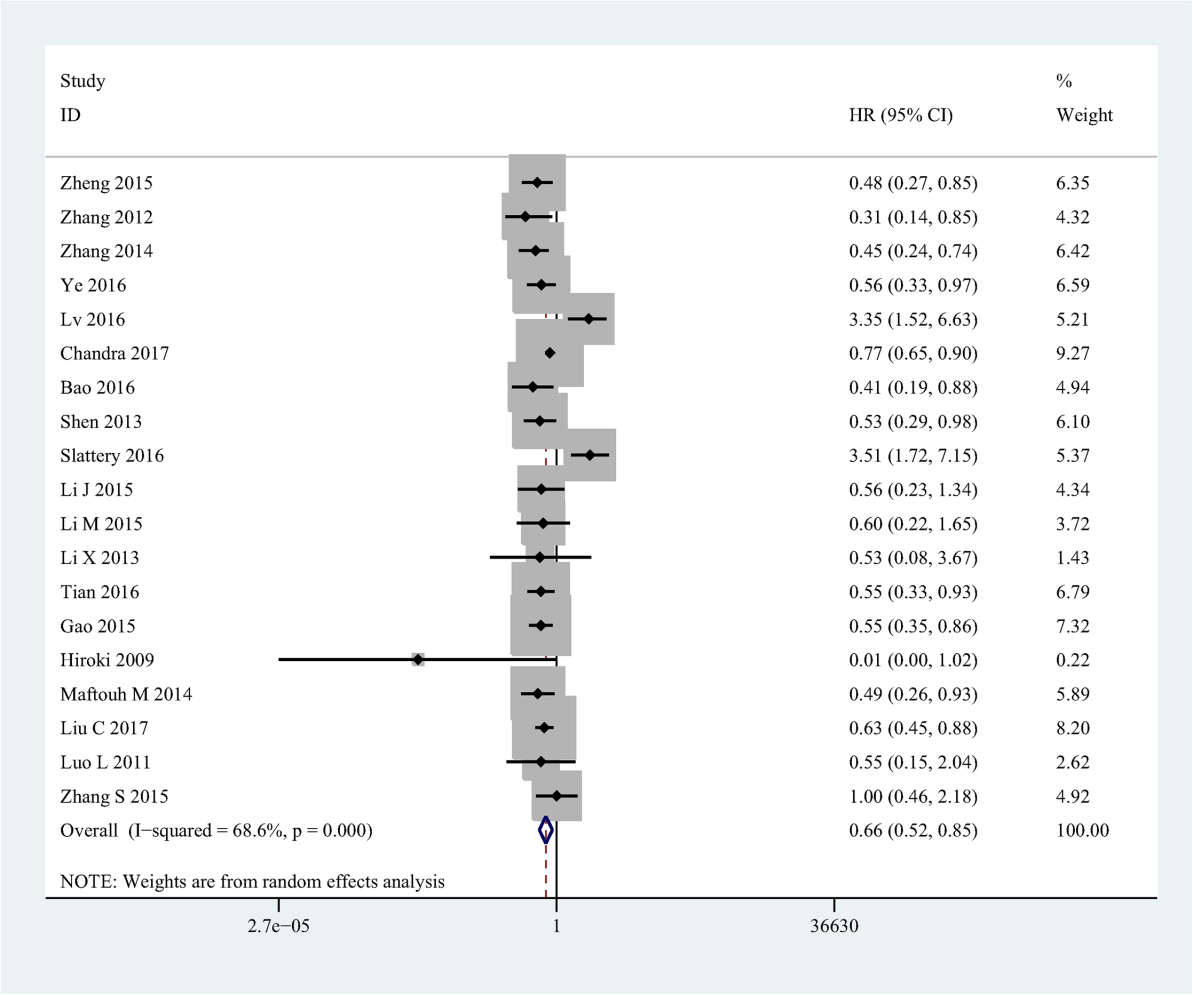
Forest plot of the relationship between miR-101 and OS in solid tumor.

Highly significant heterogeneity (I^2^ = 68.6%, p<0.001) was detected when 19 studies were pooled. To make a conservative estimate, a random-effect model rather than a fixed-effect model was used to account for the highly significant inter-study heterogeneity. The pooled HR (HR = 0.66, 95%CI [0.52–0.85], P = 0.001) suggested that lower expression level of miR-101 significantly predicted poorer OS in patients with solid tumor **(Fig 2)**.

Given that the substantial heterogeneity exhibited in the trials aggregated with respect to the OS, meta-regression and subgroups analyses were conducted to explore the heterogeneity of covariates including country, tumor type, test method, cutoff, extracting method, multivariate analysis **(Table 2)**. Subgroups analysis by country explored that lower miR-101 expression status was identified as a worse prognostic marker in China group (HR = 0.60, 95%CI [0.51–0.70], I^2^ = 49.9%, P = 0.014), but not in non-China group. According to subgroups of different cancer types, the subgroups (HCC & CRC) that significant heterogeneity was found show no significant HR(HCC HR = 0.0.72, 95%CI[0.28–1.86], I^2^ = 87.1%, P<0.001; CRC HR = 1.22, 95%CI[0.40-.67], I^2^ = 89.3%, P<0.001, fixed-effects model), which was completely opposite to BC & NSCLC & other group(BC HR = 0.76, CI[0.65–0.89], I^2^ = 0, P = 0.486; NSCLC HR = 0.56, 95%CI[0.34–0.93], I^2^ = 0, P = 0.976; Other HR = 0.54, 95%CI[0.43–0.67], I^2^ = 0, P = 0.692); With respect to subgroups by different test methods, both significant heterogeneity and HR were found in qRT-PCR group(HR = 0.61, 95%CI[0.49–0.77], I^2^ = 56%, P = 0.003), higher heterogeneity and no significant HR was reported in Microarray group (HR = 1.35, 95%[0.21–8.62], I^2^ = 93.6%, P<0.001); Subgroup analysis by Cutoff indicating that both Median (HR = 0.73, 95%CI[0.63–0.85], I^2^ = 15.9%, P = 0.312) and Other (HR = 0.61, 95%CI[0.43–0.86], I^2^ = 66.7%, P = 0.001) groups predict poor prognosis with under-expression of miR-101, in contrast, the Average (HR = 1.33, 95%CI[0.23–7.83], I^2^ = 93.5%, P<0.001) group with significant heterogeneity shows no significant HR. In the subgroup analysis based on extracting methods, The Report group (HR = 0.73, 95%CI [0.52–1.02], I^2^ = 79.4%, P<0.001) with significant heterogeneity found no significant HR, on the contrary, the K-M group (HR = 0.54, 95%CI [0.40–0.72], I^2^ = 0, P = 1) with significant HR found no significant heterogeneity. Similar to test method subgroups analysis, both groups show relative high heterogeneity in the subgroup analysis of multivariate analysis, significant HR was only found in Yes group (HR = 0.62, 95%CI [0.43–0.90], I^2^ = 70.7%, P<0.001).

**Table 2:** Pooled HRs for OS according to subgroup analysis.

Subgroup		NO. of studies	Heterogeneity		P-value	HR(95%CI)	Meta-regression	
			I^2^	P-value			adj R^2^	P-value
**Country**							7.02%	0.260
	China	15	49.9%	0.014	<0.001	0.60(0.51–0.70)^b^		
	Non-China	4	68.6%	<0.001	0.850	0.91(0.36–2.32)^a^		
**Tumor type**							-8.15%	0.614
	HCC	4	87.1%	<0.001	0.495	0.72(0.28–1.86)^a^		
	BC	2	0	0.486	0.001	0.76(0.65–0.89)^b^		
	CRC	3	89.3%	<0.001	0.730	1.22(0.40–3.67)^a^		
	NSCLC	2	0	0.976	0.024	0.56(0.34–0.93)^b^		
	Other	8	0	0.692	<0.001	0.54(0.43–0.67)^b^		
**Test method**							13.12%	0.123
	qRT-PCR	17	56.6%	0.003	<0.001	0.61(0.49–0.77)^a^		
	Microarray	2	93.6%	<0.001	0.750	1.35(0.21–8.62)^a^		
**Cutoff**							-10.08%	0.849
	Median	6	15.9%	0.312	<0.001	0.73(0.63–0.85)^b^		
	Average	2	93.5%	<0.001	0.751	1.33(0.23–7.83)^a^		
	Other	11	66.7%	0.001	0.005	0.61(0.43–0.86)^a^		
**Extracting method**							-0.79%	0.393
	Report	12	79.4%	<0.001	0.068	0.73(0.52–1.02)^a^		
	K-M	7	0	1	<0.001	0.54(0.40–0.72)^b^		
**Multivariate method**							-8.02%	0.637
	Yes	10	70.7%	<0.001	0.011	0.62(0.43–0.90)^a^		
	No	9	68.6%	<0.001	0.087	0.72(0.50–1.05)^a^		
**Overall**		19	68.6%	<0.001	0.001	0.66(0.52–0.85)		

Note

a) random-effects model

b) fixed-effects model.

### Correlation of miR-101 expression with DFS/PFS/RFS/MFS/TTP

**Fig 3:**
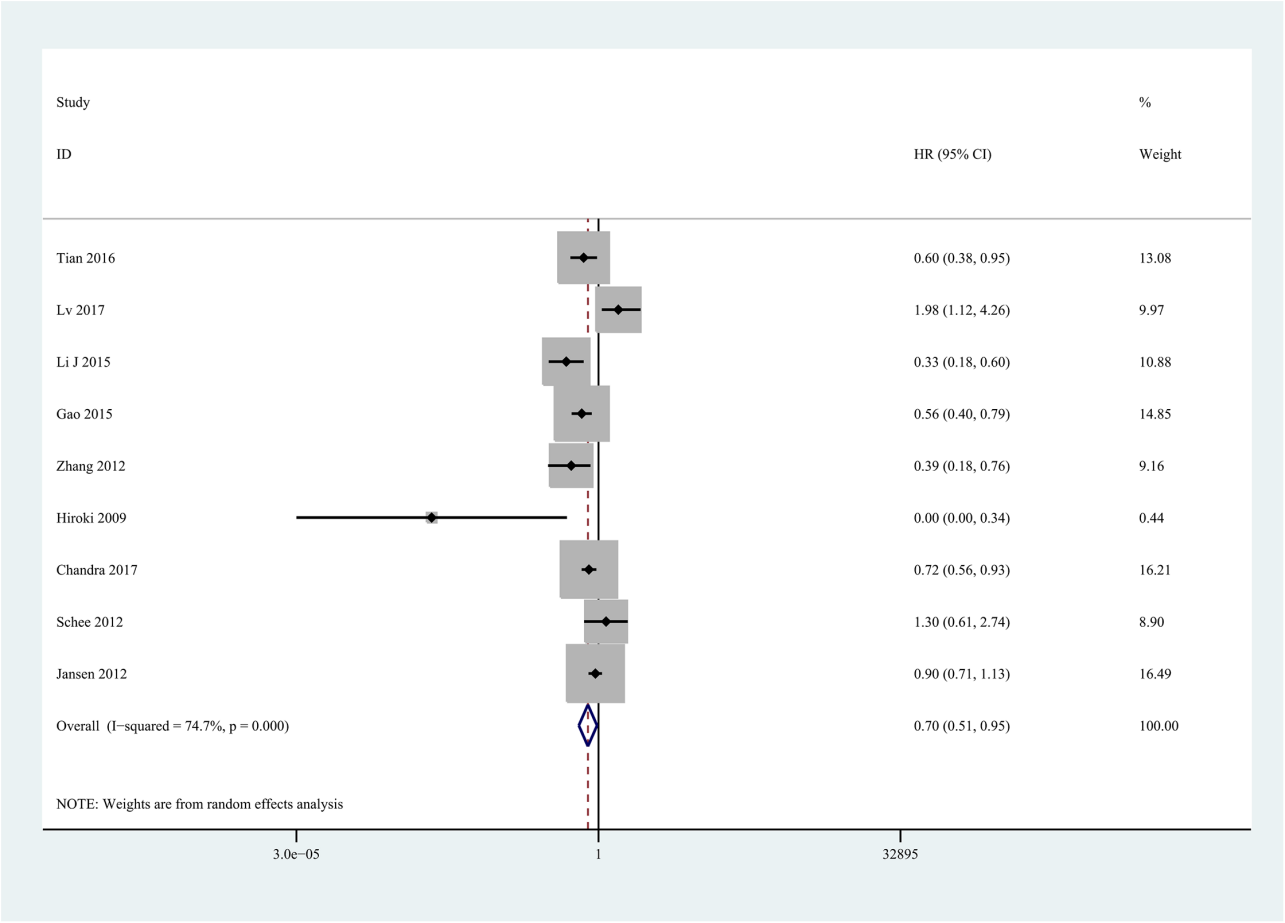
Forest plot of the relationship between miR-101 and DFS/PFS/RFS/MFS/TTP in solid tumor.

Nine eligible studies were adopted to pool HRs for DFS/PFS/RFS/MFS/TTP. With obvious statistical heterogeneity (I^2^ = 74.7%, P<0.001) (**Fig 3**), a random effect model was used to pool HRs. The result showed that low miR-101 expression was associated with negative outcome in patients with solid tumor (HR = 0.70, 95%CI [0.51–0.95], P = 0.023). Additionally, data were analyzed based on DFS, MFS, and Other. Patients with low miR-101 expression had a significantly shorter DFS (HR = 0.47, 95%CI [0.35–0.62], I^2^ = 57.1%, P = 0.072, fixed-effect model) and MFS (HR = 0.76, 95%CI [0.60–0.97], I^2^ = 53.1%, P = 0.144, fixed-effect model). Despite the lack of significant difference, a similar trend was observed for Other group (HR = 0.96, 95% [0.58–1.61], I^2^ = 76.2%, p = 0.015, random-effect model).

### Sensitivity analysis

**Fig 4:**
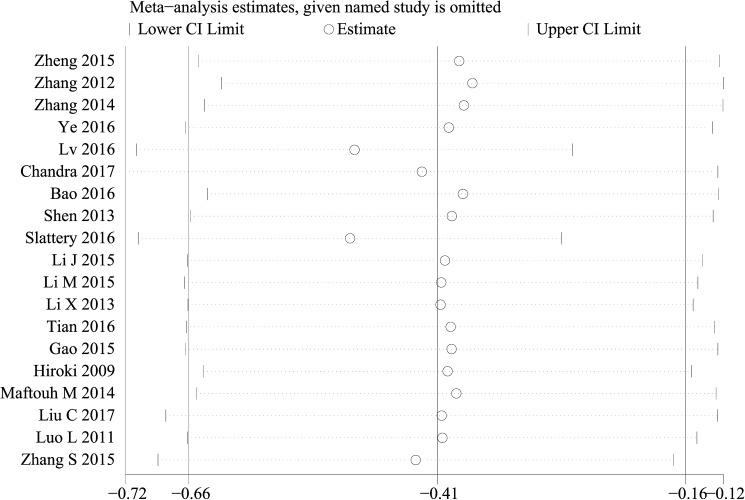
Sensitivity analysis of the evaluation on the relationship between miR-101 and OS.

**Fig 5:**
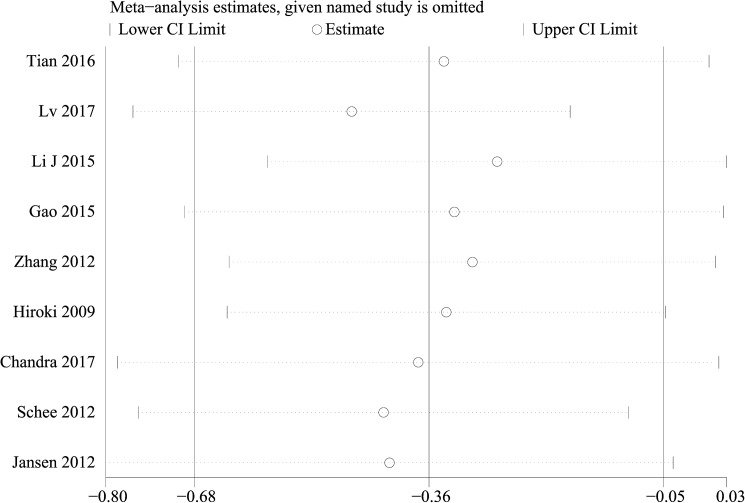
Sensitivity analysis of the evaluation on the relationship between miR-101 and DFS/PFS/RFS/MFS/TTP.

Sensitivity analysis was performed by sequentially eliminating individual studies, indicating that there was not a single study that significantly contributed to heterogeneity both for OS **(Fig 4)** and DFS/PFS/RFS/MFS/TTP **(Fig 5)**. Furthermore, a meta-regression was also conducted to explore the potential factors that are responsible for heterogeneity in OS, The results showed that the above factors could partly explain the heterogeneity but did not reach statistical significance **(Table 2)**.

### Publication bias

**Fig 6:**
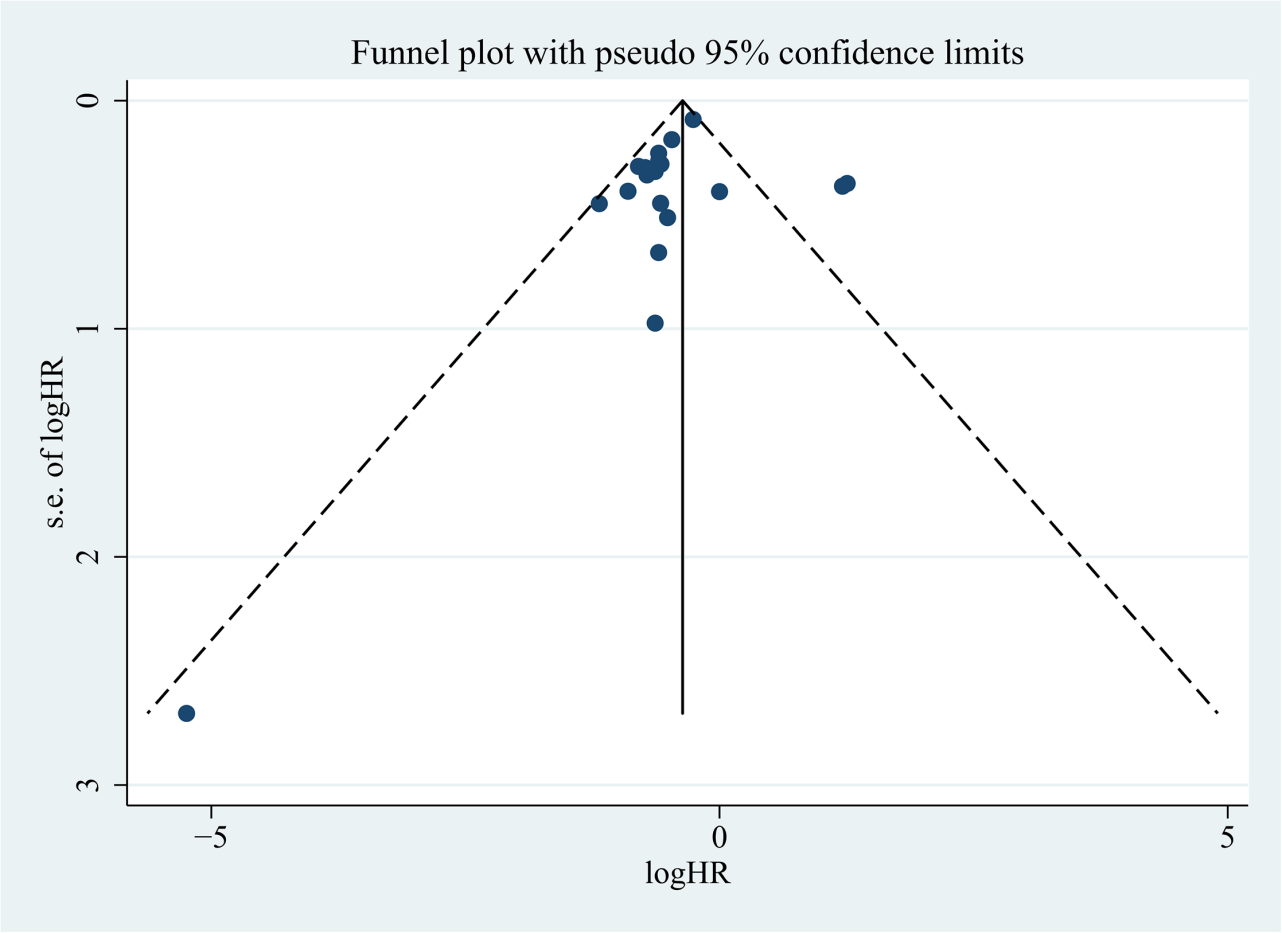
Funnel plots to evaluate publication bias of included studies for OS.

**Fig 7:**
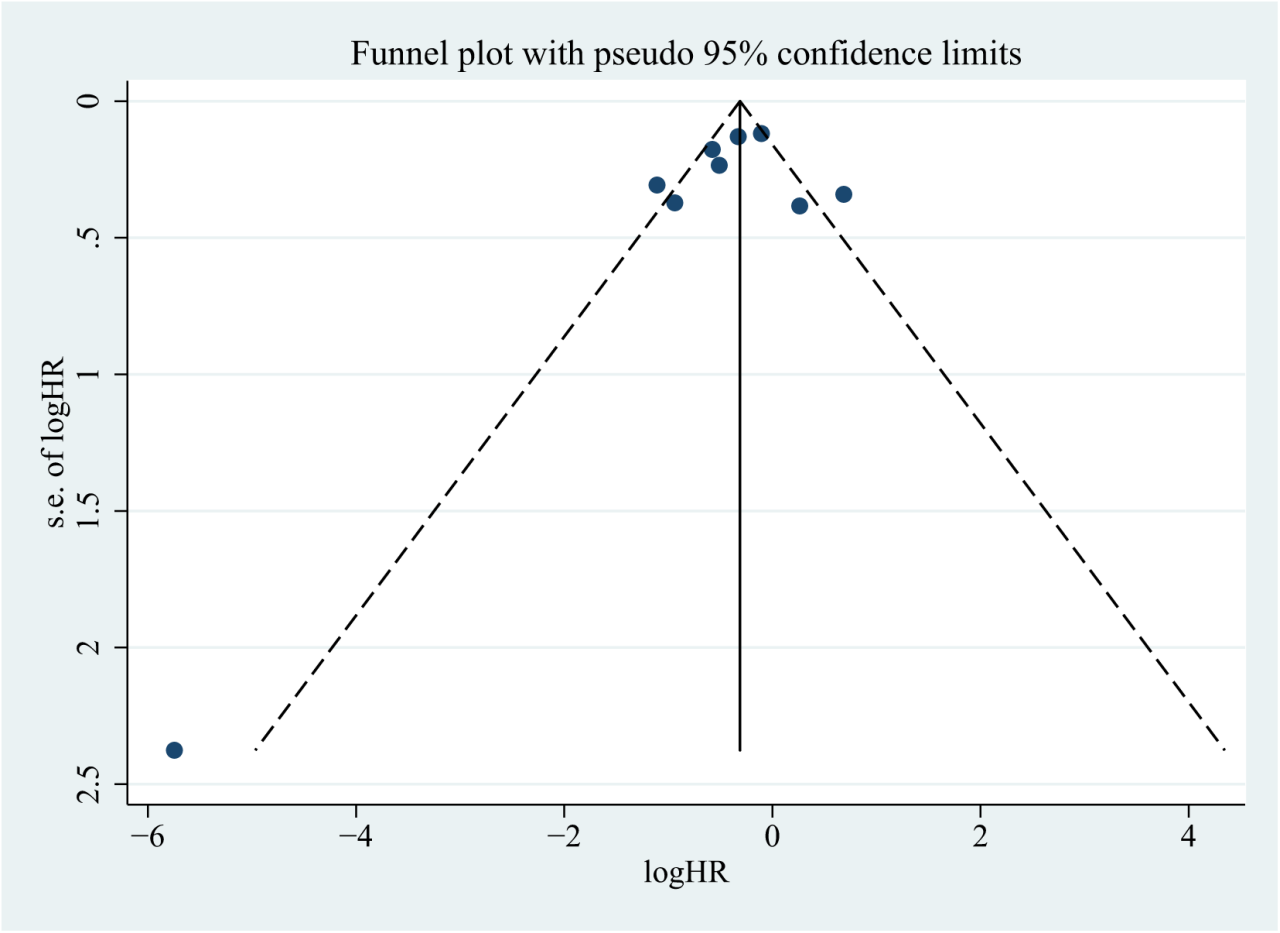
Funnel plots to evaluate publication bias of included studies for DFS/PFS/RFS/MFS/TTP.

The Begg’s funnel plots, Egger’s test and Begg’s test were used to detect publication bias in the meta-analysis. Although the funnel plot revealed relative big publication bias in OS, but P-value of Begg’s and Egger’s tests were 0.861 and 0.166, respectively, showing no evidence for significant publication bias **(Fig 6)**. Similarly, P-value of Begg's and Egger's tests for DFS/PFS/RFS/MFS/TTP were 0.297 and 0.765, no significant publication bias was detected, either (**Fig 7**).

## Discussion

Numerous profiling studies have demonstrated that miRNA expression levels failed to agree in various types of cancers, miRNAs can be potential biomarkers for cancer prognosis. Increasing data favor the potential use of miR-101 as a cancer prognostic predictor. Recently, genome-wide miRNA expression profiling studies revealed that miR-101 is widely present in various tissues and organs, and its aberrant expression was reported in various cancers including HCC[14, 19, 20, 25], CRC[13, 30], breast cancer[23, 26], NSCLC[22], gliomas[28], and et al. It is indubitable that miR-101 is an important cancer-related miRNA. More and more evidence has demonstrated that miR-101 is frequently downregulated in multiple types of cancer and acts as a tumor suppressor by repressing many critical oncogenes. In hepatocellular carcinoma, Wang et al found c-Myc collaborates with EZH2-containing PRC2 complex in silencing miRNA-101 during hepatocarcinogenesis and lower expression of miR-101 is positively correlated with poorer prognosis [38]. In CRC, it is reported that loss of miR-101 expression promotes Wnt/β-catenin signaling pathway activation and malignancy in colon cancer cells[39]. Similarly, in glioblastoma, Liu et al demonstrated that miRNA-101 inhibits proliferation, migration and invasion of glioblastoma by targeting SOX9 [40], Michiel et al also found that miRNA-101 is down-regulated in glioblastoma resulting in EZH2-induced proliferation, migration, and angiogenesis [41]. Moreover, miRNA-101 reverses temozolomide resistance by inhibition of GSK3β in glioblastoma[42]. In nasopharyngeal carcinoma, MicroRNA-101 inhibits invasion and angiogenesis through targeting ITGA3 and its systemic delivery inhibits lung metastasis[43]. On the other hand, 175 targeted genes validated by experiment and 5206 targeted genes predicted by miRanda software can be found in GCBI website based on classical miRNA-3’-UTR pathway (https://www.gcbi.com.cn/gclib/html/dictSearchAct/MI0000103/miRNA), indicating miRNA-101 may play a complicated role in many gene ontology functions and pathways networks. Further experiments need to be conducted to elucidate the role of miR-101 in carcinogenesis. However, among all the studies referring to the relationship between miRNA-101 and OS/PFS, there were still some contradictory views requiring adequate attention, a comprehensive study is therefore in urgent.

To the best of our knowledge, few studies have systemically explored the possible prognostic role of miR-101 down-regulation in solid malignancies before. In order to get a more convincing outcome, HRs for both OS and PFS were calculated independently. On the whole, our quantitative results strongly supported the current mainstream viewpoint that an undesirable impact of miR-101 low expression was related with poor OS and PFS, taking no account of confounding factors. Among all the included studies, on the contrary, two studies [13, 14] highlighted that obvious advantage on survival duration was obtained in miR-101 under-expression cases and no significant HR was found in other three studies [26–28, 35], whose HR value and 95%CI were extracted from K-M survival curve, indicating that this indirect method may impose slightly bias on the HR we calculated. It’s worth mentioning that 4 studies providing K-M curve was excluded due to the lack of clear categorization [9, 44–46]. The appropriate HRs can't be obtained until the K-M survival curve and the exact number of each group are available simultaneously. Based on the outcome of subgroup analysis, significant HR was not found in all subgroup even significant HR was found in each studies, which may partly attribute to a relative small number of studies and high heterogeneity and need further elucidate.

Apart from the inspiring outcomes, limitations still exist in this quantitative meta-analysis. First of all, despite the usage of random-effects model and subgroup analysis, the heterogeneity across studies failed to be eliminated completely, which could result in bias of the outcome in certain extent. Secondly, due to lack of direct HR and 95%CI data, we merely extracted the data by using Engauge software indirectly, which may bring about slight error in HR and 95%CI. Thirdly, lack of abundant miR-101 expression data in the global population makes it difficult to set a standard cut-off value for measurement of miR-101 expression levels, categorization between studies did not get in consensus. Additionally. Other parameters that may partially contribute to the heterogeneity were not explored, such as pathological grade and body mass index.

In spite of the limitations mentioned above, there are still numerous valuable implications in this comprehensive meta-analysis, which reveals that low expression of miR-101 is associated with unfavorable survival outcomes in patients with various types of carcinomas, particularly with regard to OS. Further large-scale, well-designed and multi-center prospective studies should be conducted to confirm these findings before the application of miR-101 for the prognosis of cancers.

## Supporting information

S1 Fig Forest plot to assess the association between miR-101 under-expression and OS in subgroups based on test method. (TIF)

S2 Fig Forest plot to assess the association between miR-101 under-expression and OS in subgroups based on extracting method. (TIF)

S3 Fig Forest plot to assess the association between miR-101 under-expression and OS in subgroups based on country origin. (TIF)

S4 Fig Forest plot to assess the association between miR-101 under-expression and OS in subgroups based on multivariate method. (TIF)

S5 Fig Forest plot to assess the association between miR-101 under-expression and OS in subgroups based on tumor type. (TIF)

S6 Fig Forest plot to assess the association between miR-101 under-expression and OS in subgroups based on cutoff method. (TIF)

S1 Table PRISMA 2009 checklist. (DOC)
