# Supplementary figures and images for: Prognostic significance of microRNA-101 in solid tumor: A meta-analysis
Source: PLoS One. 2017 Jul 25;12(7):e0180173. doi: 10.1371/journal.pone.0180173 (PMC5526582; doi:10.1371/journal.pone.0180173)

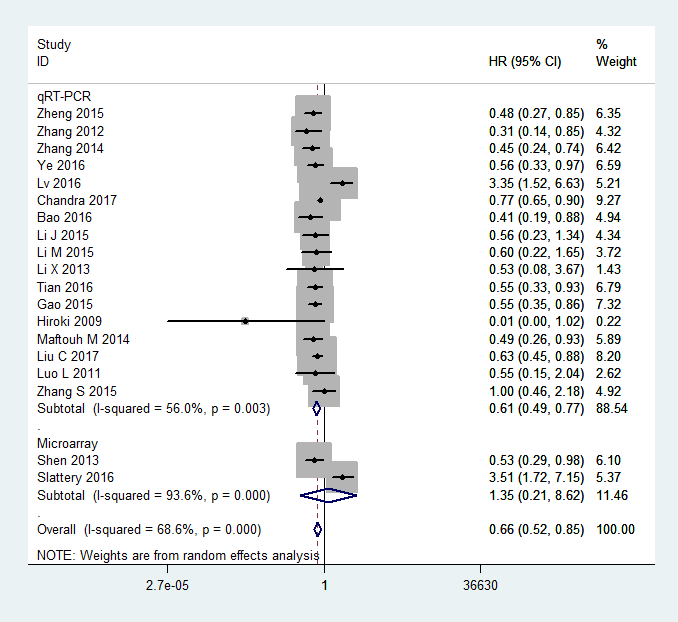

Supplement: S1 Fig — (TIF) [file pone.0180173.s001.tif]

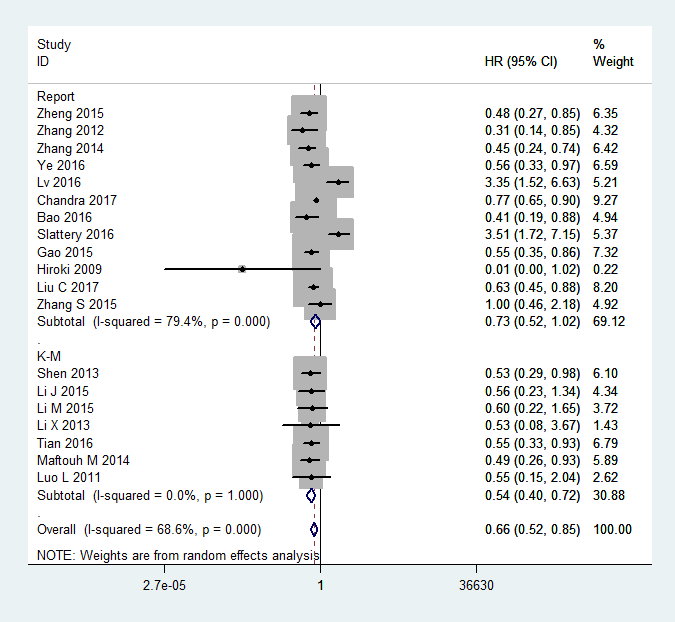

Supplement: S2 Fig — (TIF) [file pone.0180173.s002.tif]

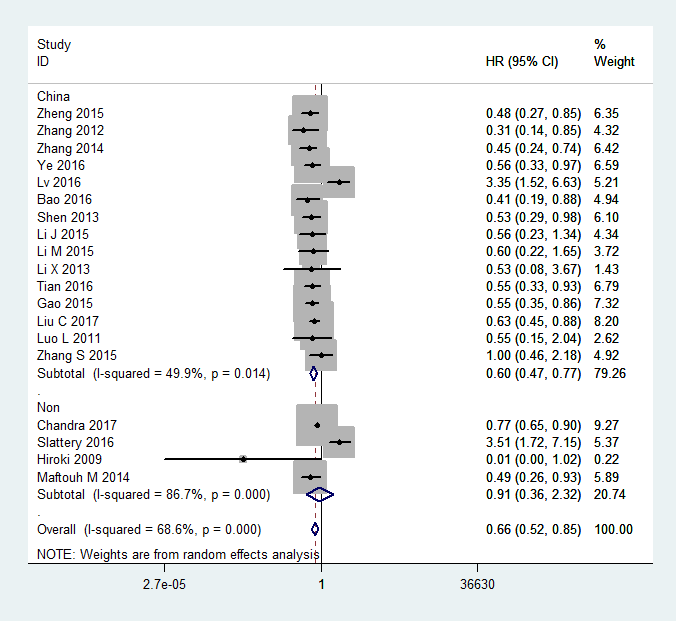

Supplement: S3 Fig — (TIF) [file pone.0180173.s003.tif]

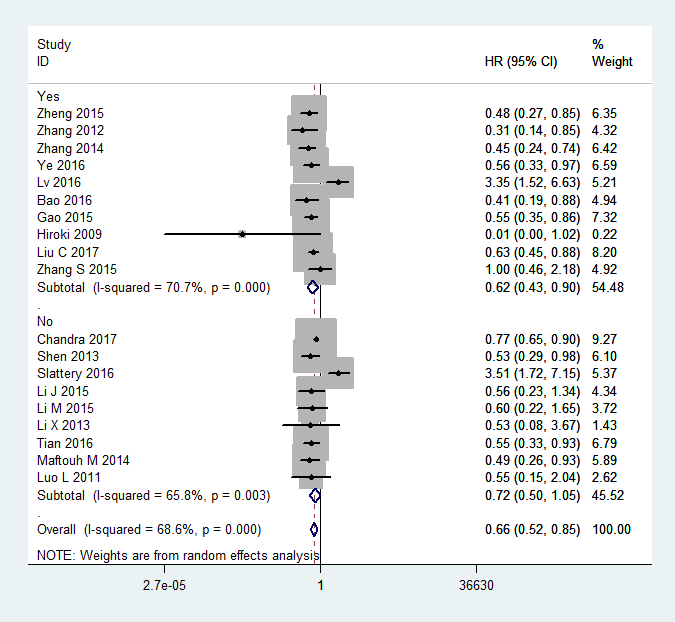

Supplement: S4 Fig — (TIF) [file pone.0180173.s004.tif]

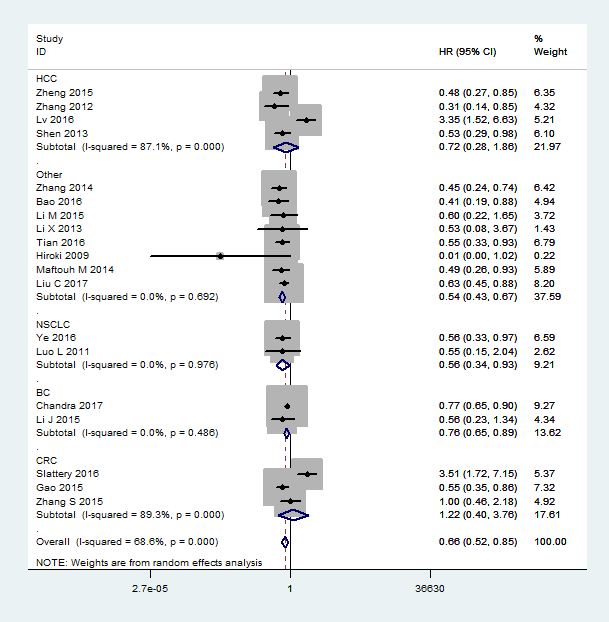

Supplement: S5 Fig — (TIF) [file pone.0180173.s005.tif]

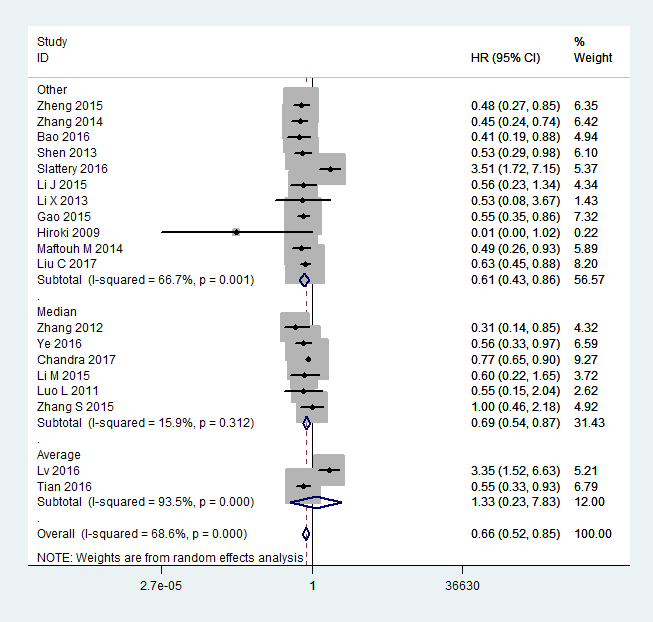

Supplement: S6 Fig — (TIF) [file pone.0180173.s006.tif]
